# Why Verbalization of Non-Verbal Memory Reduces Recognition Accuracy: A Computational Approach to Verbal Overshadowing

**DOI:** 10.1371/journal.pone.0127618

**Published:** 2015-06-10

**Authors:** Aya Hatano, Taiji Ueno, Shinji Kitagami, Jun Kawaguchi

**Affiliations:** 1 Department of Psychology, Graduate School of Environmental Studies, Nagoya University, Nagoya, Japan; 2 Psychology Department, University of York, York, United Kingdom; 3 Japan Society for the Promotion of Science, Tokyo, Japan; Bournemouth University, UNITED KINGDOM

## Abstract

Verbal overshadowing refers to a phenomenon whereby verbalization of non-verbal stimuli (e.g., facial features) during the maintenance phase (after the target information is no longer available from the sensory inputs) impairs subsequent non-verbal recognition accuracy. Two primary mechanisms have been proposed for verbal overshadowing, namely the recoding interference hypothesis, and the transfer-inappropriate processing shift. The former assumes that verbalization renders non-verbal representations less accurate. In contrast, the latter assumes that verbalization shifts processing operations to a verbal mode and increases the chance of failing to return to non-verbal, face-specific processing operations (i.e., intact, yet inaccessible non-verbal representations). To date, certain psychological phenomena have been advocated as inconsistent with the recoding-interference hypothesis. These include a decline in non-verbal memory performance following verbalization of non-target faces, and occasional failures to detect a significant correlation between the accuracy of verbal descriptions and the non-verbal memory performance. Contrary to these arguments against the recoding interference hypothesis, however, the present computational model instantiated core processing principles of the recoding interference hypothesis to simulate face recognition, and nonetheless successfully reproduced these behavioral phenomena, as well as the standard verbal overshadowing. These results demonstrate the plausibility of the recoding interference hypothesis to account for verbal overshadowing, and suggest there is no need to implement separable mechanisms (e.g., operation-specific representations, different processing principles, etc.). In addition, detailed inspections of the internal processing of the model clarified how verbalization rendered internal representations less accurate and how such representations led to reduced recognition accuracy, thereby offering a computationally grounded explanation. Finally, the model also provided an explanation as to why some studies have failed to report verbal overshadowing. Thus, the present study suggests it is not constructive to discuss whether verbal overshadowing exists or not in an all-or-none manner, and instead suggests a better experimental paradigm to further explore this phenomenon.

## Introduction

Verbal overshadowing refers to a detrimental effect that verbalization has on accuracy/reaction time performance in non-verbal memory tasks (e.g., for faces, voices, or tastes). Schooler and Engstler-Schooler [1] first established a paradigm to investigate this phenomenon. Specifically, participants watched a video of a bank robbery for 30 seconds. After this, half of them were asked to verbalize the appearance of the bank robber, whereas the remaining half was not. Subsequently, all of the participants took an eight-alternative forced choice face recognition test (a photo line-up). Recognition performance was worse for those who had verbalized the appearance of the bank robber than those who had not. This phenomenon was termed *verbal overshadowing*. This phenomenon can be observed in daily life and in legal settings (e.g., eyewitness reports)[2]. As such, elucidation of the mechanisms underlying this phenomenon is significant for applied issues as well as being relevant for theories of cognition. Meissner and Brigham’s meta-analysis confirmed the robustness of this phenomenon [3], and very recently, a large-scale study has also confirmed its robustness [4]. Typically, the incorrect responses occur as more frequent selections of a distractor face (i.e., false alarms), rather than as “not present” responses in N-alternative forced choice recognition tasks [5]. Thus, the nature of distractors modulates the effect of verbalization. More specifically, verbal overshadowing occurs when distractors are relatively similar to the target face [6]. Related to this, if a target is a typical face (thus, more similar to distractors), verbal overshadowing is more readily observed [7].

Verbal overshadowing is observed for various kinds of non-verbal stimuli, including facial images [8], voices [9,10], and tastes [11,12]. In the present study, we use the term verbal overshadowing in a narrow sense to refer to non-verbal memory performance impaired by verbalization. Other studies sometimes use this term more extensively beyond the meaning as per the original memory studies, including to refer to reduced accuracy in insight problems [13] or analogies [14], and so on. Of course, it is tempting and might be parsimonious for a single theory to explain the similar findings across domains. However, any attempt to develop a theory within a domain (i.e., memory) should be conservatively restricted to that domain, unless there is a strong a-priori reason to extend the explanation more widely. So far, two primary explanations have been offered. The first is the *recoding interference hypothesis* [1,3], which assumes that verbalizing non-verbal memory renders the (rich and sophisticated) visual representations imperfect and less accurate. This recoded representation is assumed to be used in subsequent face recognition, resulting in a reduction in accuracy. Thus, the recoding interference hypothesis predicts that verbal overshadowing will occur more readily if participants generate less accurate verbal descriptions. This assumed process links Bruce and Young ‘s [15] face recognition model, which assumes that both visual code and verbal code affect face recognition. Consistent with the RIF account, Meissner, Brigham, and Kelley [5] found clearer verbal overshadowing when participants were prompted to generate as many descriptions as possible, no matter how accurate they thought those descriptions were. Conversely, verbal overshadowing was attenuated when the participants were warned not to generate a description unless they were confident with its preciseness. These relationships were further supported by positive subject wise correlations between recognition accuracy and accuracy of the verbal descriptions. Related to this, Meissner and Brigham [3], in their meta-analysis, also demonstrated that verbal overshadowing is liable to occur when participants verbalize a target in detail. The authors explained that a detailed description more frequently elicits inaccurate verbal descriptions, and these inaccurate descriptions in turn create an inaccurate (recoded) representation.

In contrast, the other proposed mechanism underlying verbal overshadowing is the *transfer-inappropriate processing shift (TIPS) hypothesis* [16–19], which proposes that verbalization requires a *shift* into verbal processing, and this shift obstructs the application of non-verbal (face-specific) processing in the subsequent face recognition phase. Like the transfer-appropriate processing account of memory[20], the TIPS hypothesis assumes that non-verbal stimuli should be best recognized in non-verbal, face-specific processing operations, such as configural/holistic processing [21,22]. Specifically, in the encoding phase, a target face may be encoded into a rich and sophisticated representation by non-verbal, face-specific processing operations. However, describing the face during the retention phase may enhance verbal processing operations. Then, these verbal processing operations may be inappropriately carried over into the subsequent recognition phase. As a result, participants cannot apply face-specific processing and fail at choosing the correct target. Taken together, both theories assume that face stimuli are best represented in a non-verbal format, and such a sophisticated non-verbal representation is necessary for face recognition [21]. However, the two accounts differ in terms of the status of such a non-verbal representation after verbalization. The recoding interference hypothesis assumes that verbalization renders the representation itself less accurate, whereas the TIPS hypothesis assumes that the non-verbal representation is unavailable. Thus, the latter assumes that verbalization requires a shift in the processing itself, with the non-verbal representation itself remaining intact [23], but this shift obstructs access to the intact non-verbal representation.

So far, both supporting evidence and counterevidence has accumulated from human experiments for both theories. For example, Schooler [19] pointed out that the recoding interference hypothesis cannot explain some results that TIPS can. For example, Dodson, Johnson, and Schooler [23] demonstrated that verbalization of a non-target face (e.g., a parent’s face) also impaired recognition of the target face [17,18,24]. If an inaccurate target representation made by verbalization is responsible for memory impairments (i.e., the recoding interference hypothesis), verbalizing an unrelated face would not affect subsequent recognition. Additional evidence against the recoding interference hypothesis is that some studies have failed to replicate a positive correlation between recognition accuracy and the verbal description accuracy of the target face [6,8,25]. Consequently, Schooler [19] has argued that a crucial aspect of verbal overshadowing is not the inaccurate verbal representation, but a processing shift that in turn obstructs the re-application of non-verbal operations. Thus, it is worth emphasizing that the TIPS has a more modularistic relationship (as exemplified by terms such as “shift” and “apply”) with non-verbal and verbal processing than the recoding interference hypothesis. The TIPS hypothesis clearly assumes an *operation-specific (process-specific) representation*, and argues this can accommodate the behavioral data.

These experimental data have greatly contributed to testing the competing theories. The next step is to bring these informal (i.e., descriptive) theories into an engineering framework by taking a computational modeling approach. The rationale behind this approach is that if a theory is correct, then a computational model that instantiates the core processing principles of that theory should reproduce the relevant phenomena. Moreover, given the experimental evidence against the recoding interference hypothesis (above), it is theoretically meaningful to simulate such “counterevidence” in a working computational model of the recoding interference hypothesis (meaning it is no longer counterevidence), which can trigger a more thorough debate between two theories.

Finally, the present computational model also addressed the replicability issues in verbal overshadowing. Our hypothesis is that this is due to (a) the use of single-trial testing methods, (b) individual differences, and (c) relatively uncontrolled extraneous variables (e.g., how many distinctive facial features the target and distractors share). These issues will be addressed following the main simulation experiments.

## Method

### Ethics statement

We did not collect any human data. This work takes a computational modelling approach to simulate human behavioral data, which were taken from existing literature.

### Model architecture

**Fig 1:**
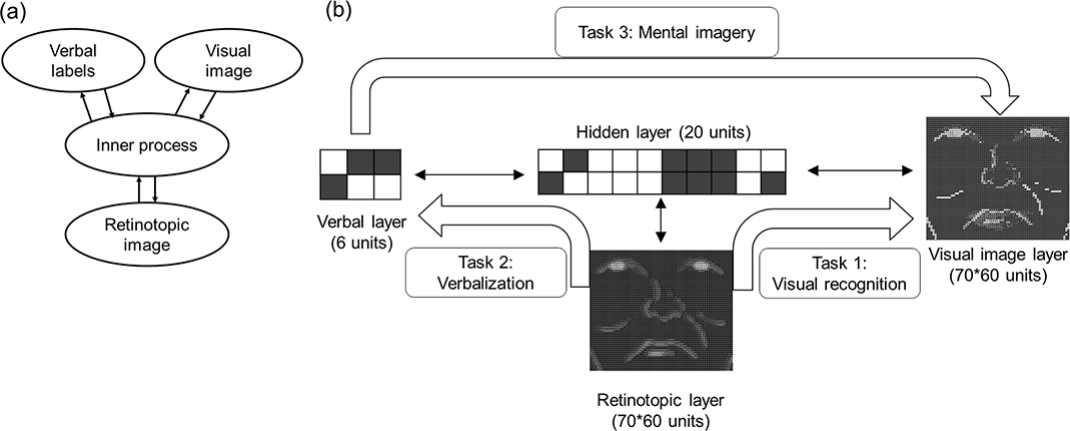
An abstract connectionist framework for face processing (left) and an actual model architecture (right). The number of units in each layer is indicated in parentheses. Each solid bi-directional arrow represents connectivity from one group of units to another.

The PDP model was built using the Light Efficient Neural Simulator (LENS) software (http://tedlab.mit.edu/~dr/Lens/). Fig 1 shows an abstract connectionist framework for face processing (left) and the actual model architecture (right). The retinotopic layer (4,200 units) received a noise-filtered face input. Then, the activations spread to the other layers, and a clear (i.e., non-filtered) face image was represented in the visual image layer (upper-right layer, 4,200 units), whereas verbal information of the face features was represented in the verbal layer (6 units). The details of the tasks and mappings will be explained later. These three peripheral layers were connected bi-directionally through a single hidden layer (20 units). In order to reduce the computational demands in this large model, units in a peripheral layer were connected to the hidden layer only when the external input/target value of that unit had variations (i.e., sparse connectivity).

### Stimuli (Representations)

**Fig 2:**
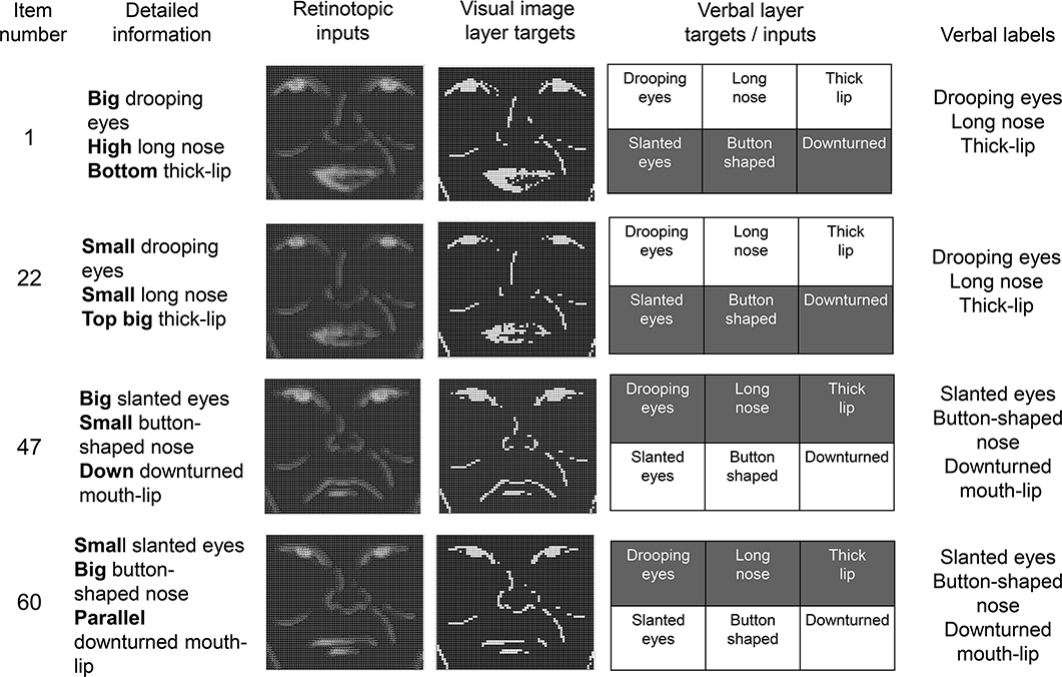
Four example items. **The 3** ^**rd**^ **through 5** ^**th**^ **columns show the representations (Hinton diagrams) in each layer.** The leftmost column shows the item ID numbers for each face pattern. The second column shows the unique combination of the specific (shown in bold) features of each face, which the model could not discriminate by the activation patterns of the verbal layer (i.e., 5^**th**^ column). The third column shows the retinotopic patterns of each item. The fourth column shows the target patterns of the visual image layer. The fifth and sixth columns show the activation patterns (and their labels) in the verbal layer.

We will first explain how the 64 face items (image files) were selected. Then, we will describe how we created the representations (vector patterns) of these 64 items that a computational model received/generated (Fig 2 shows four examples of these 64 items). Specifically, we first selected two verbal labels for each of the three face features (i.e., *drooping/slanted eyes*; *long/button-shaped noses*; *thick/downturned lips*; see the 6^th^ column of Fig 2). Then, for each of these six verbal labels, two subordinate types were created (i.e., big/small *slanted eyes*, big/small *drooping eyes*; big/small *long nose*, big/small *button-shaped nose*; bottom/top *thick lip*, normal/parallel *downturned lip*, see the 2^nd^ column of Fig 2). In order to make the model trainable, we deliberately kept the stimuli as simple as possible, by excluding other distinctive features such as hair. By crossing these four specific types of eyes/noses/lips, 64 (i.e., 4 × 4 × 4) unique face patterns were formed.

Next, the representations (vector patterns) in each layer for these 64 items were created via the following steps. First, visual image files for the 64 faces (70 × 60 pixels) were created (see S1 Fig) using montage software (http://www1.mahoroba.ne.jp/~matumoto/nitaroS.html). Then, each of these image files was binarized (i.e., a black pixel was 1 and a white pixel was 0), forming a 4,200-bit vector pattern. These were used as the targets for the visual image layer (see the 4^th^ column of Fig 2). Next, in order to create the input patterns of these items to the retinotopic layer (3^rd^ column of Fig 2), the images were smoothed by Gaussian convolution (*SD* = 0.2, see S2 Fig). It is still debatable which noise function would be the best to represent noisy retinotopic map [26]. Therefore, we simply followed the existing literature [27]. Finally, as shown in the 5^th^ and 6^th^ columns of Fig 2, the 6-bit vector patterns in the verbal layer represented one of the six verbal labels for the face features in a localist manner (i.e., *drooping/slanted eyes*; *long/button-shaped noses*; *thick/downturned lips*), not the specific features within a verbal label (e.g., big slanted eyes). Therefore, the activation patterns in the verbal layer could not discriminate two items sharing the same verbal labels (e.g., 1^st^ and 2^nd^ rows of Fig 2).

### Tasks

#### Visual recognition task

In this task, a noise-filtered face image was presented to the retinotopic layer. Then, the model had to map this retinotopic input (third column of Fig 2) into a non-filtered, item-specific visual representation in the visual image layer (fourth column of Fig 2). Thus, like past computational models [27,28], face recognition was simulated in terms of whether item-specific information was computed from a noisy input. In each trial, the network was allowed to cycle 10 time steps. At each step, the activation spread to the next layer, gradually being scaled by the values of the interconnecting weights. The net input was computed by considering the net input at the previous time step, using the following equations:

$$s_{i.t}=s_{i.t-1}+0.1*\left(\sum_{j}w_{j.i}•a_{j.t}-s_{i.t-1}\right)$$ (1)

$$a_{i.t}=\frac{1}{1+e^{-s_{i.t}}}$$ (2)

Where *s*_*i*.*t*_ is the net input at time step *t* from all projections *j* to the unit *i*. *a*_*i*.*t*_ is the activation of the units *i* at time step *t*, which is a logistic function of the net input, ranging from 0 to 1.

The target, correct activation value of each unit, was assigned to the visual image layer, and the output pattern in this layer was compared to this target pattern from the first cycle. If the output-target difference dropped below 0.5 for all units in the visual image layer, then the next trial (next face) began, even if this was before the 10^th^ cycle. Error (difference between the output and the target) of the target units was estimated by a cross entropy method [29].

#### Verbalization task

In this task, the model received the same retinotopic input as the visual recognition task (see above), but was required to activate the correct units of the verbal layer (i.e., correct verbal labels, see 5^th^ column of Fig 2). As Fig 2 shows, the model had to activate three units, each of which corresponded to the eyes/nose/lip labels, respectively. Like the visual recognition task, the model was allowed to cycle 10 steps in each trial. The training for each trial was terminated according to the same criterion as the visual recognition task.

#### Mental imagery task

In this task, the verbal layer received an external input, and the visual image layer activity was compared to its target pattern. The model had to compute the correct non-filtered, face image from the verbal labels. The retinotopic layer did not receive any external input. It should be noted that the accuracy in this task never reached 100% because sometimes a different target pattern would be computed from the same input pattern (i.e., one-to-many mapping). Taking the 1^st^ and 2^nd^ rows of Fig 2 as examples, the visual image layer patterns (i.e., targets) were different, but their verbal layer activations (i.e., inputs in the mental imagery task) were identical. Therefore, the same units in the verbal layer (e.g., *drooping eyes*) were activated for these two cases, but different output patterns (big or thin eyes) should be generated in the visual image layer. The model could not decide which pattern to compute in the visual image layer from the identical input pattern, as a result of which accuracy never reached 100%. This is consistent with a situation that occurs when humans imagine the appearance of a person upon hearing a verbal description of facial features. Humans also cannot specify a unique person from the various people who share the same verbal labels.

#### Training

As will be explained later, half of the 64 faces were used for training, and the remaining half was used for testing generalization. The model was first trained on the visual recognition task (pre training) until its accuracy exceeded 50% because human babies first learn visual recognition before acquiring language. Then, the training trials for the other two tasks were interleaved with the visual recognition task. After training each item 3,000 times in each of the three tasks (after the pre-training of visual recognition), the model’s performance was satisfactory (see later for details).

The connection strength was adjusted by a back-propagation through time algorithm. The model did not add previous learning outcomes (weight changes) to the current training (i.e., the momentum parameter was set to zero). If the difference between the output and the target activation value was within 0.1, then no error derivative was back-propagated from that unit. The model was trained with a learning rate of 0.05, and a decay parameter set to 0.00000001. The batch size was 1. A small amount of Gaussian noise (*SD* = 0.2) was added to the input to the hidden layer to encourage this layer to adopt more polarized outputs. These parameter values are common in the neural network literature [30,31], and we simply followed these values.

## Results

### Training performance

Five models were trained on three tasks: a visual recognition task, a verbalization task, and a mental imagery task. As mentioned above, a randomly selected half of the items (i.e., 32 items) was used during training whereas the remaining half was not. This manipulation resulted in creating 32 “old” (trained) faces for the model and 32 “new” (untrained) faces. First, the model was able to activate the correct verbal units (i.e., verbalization task) for all 32 trained and 32 untrained items (i.e., generalization occurred). With regard to the mental imagery task, this never reached 100% accuracy.

**Fig 3:**
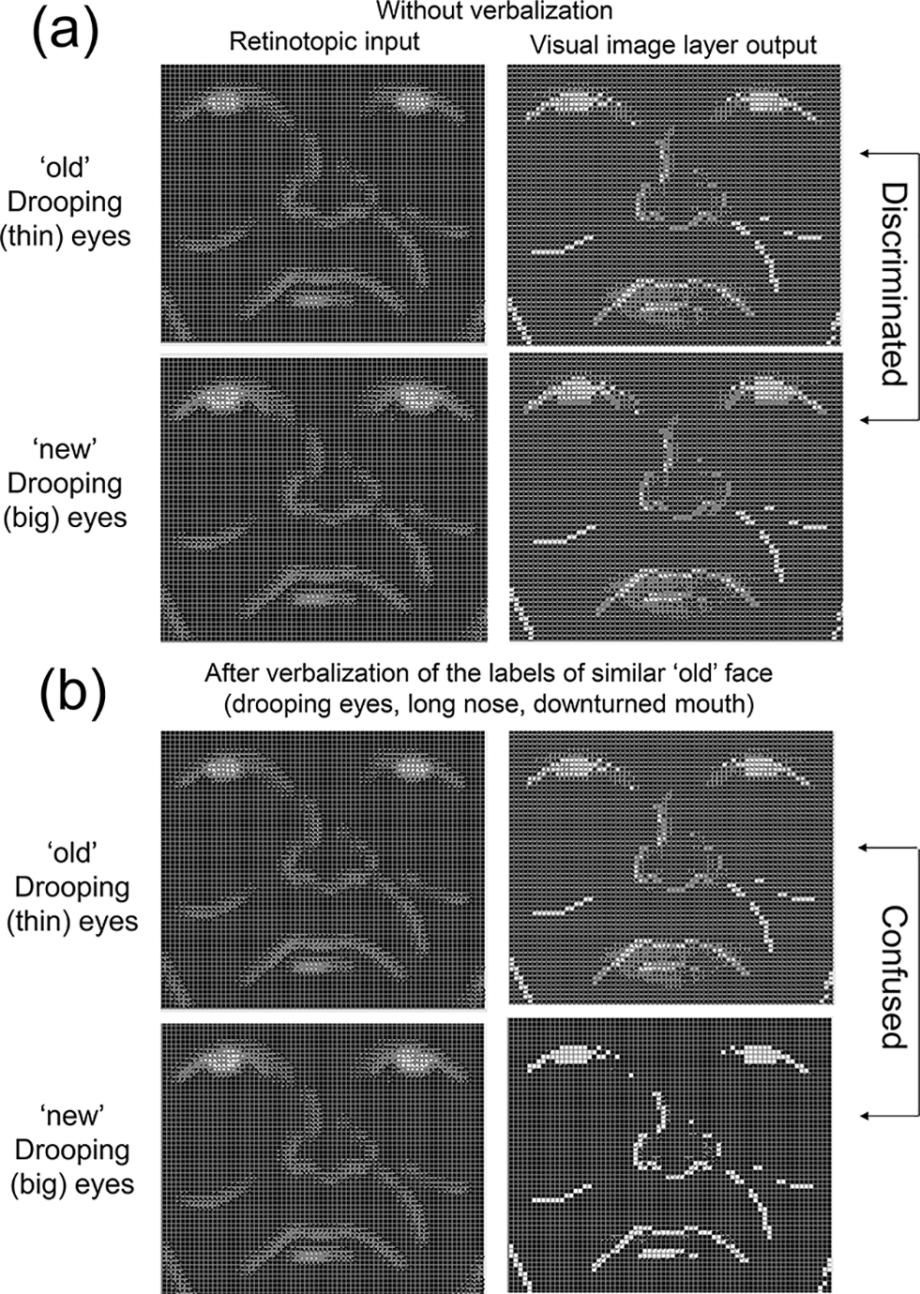
Hinton diagrams of the face recognition outcomes for two visually-similar trained/untrained faces, respectively. The diagrams in the left column denote the retinotopic input, and those in the right column denote the generated outputs in the visual image layer. The upper half (a) shows the outcomes of the face recognition without prior verbalization of facial features whereas the lower half shows those after verbalization of facial features (b).

Next, we evaluated visual recognition performance by examining whether or not the model could differentiate Person X from others. Such differentiation should indicate that the model was successfully trained to compute item-specific information for each trained/untrained item without confusing with others, and the results supported that the model was able to do this [32,33]. Fig 3A (upper half) shows two successful examples in the visual recognition task. Specifically, these two faces shared the same mouth/nose features, yet the upper, trained (i.e., “old”), case had *thin* whereas the lower, untrained (i.e., “new”), case had *big* drooping eyes. A visual inspection of the visual image layer in the right column suggests that the model was able to discriminate these two visually similar cases, and generated item-specific outputs in the layer. Furthermore, the output vector in the visual image layer was compared to all 64 target vector patterns to provide a more systematic evaluation. The rationale behind this nearest-neighborhood criterion analysis is that, if the closest vector pattern is its target vector (i.e., smallest Euclidean distance), then the model represented correct item-specific information in the visual image layer. With this scoring method, accuracy for visual recognition was 100% for the 32 trained (“old”) items and 84% (SD = 0.05%) for the 32 untrained (“new”) items. These indicated the model was computed the correct verbal and visual representations from a noisy retinotopic input of the 32 old faces and generalized the acquired knowledge to discriminate 32 untrained “new” faces from the “old” faces.

**Fig 4:**
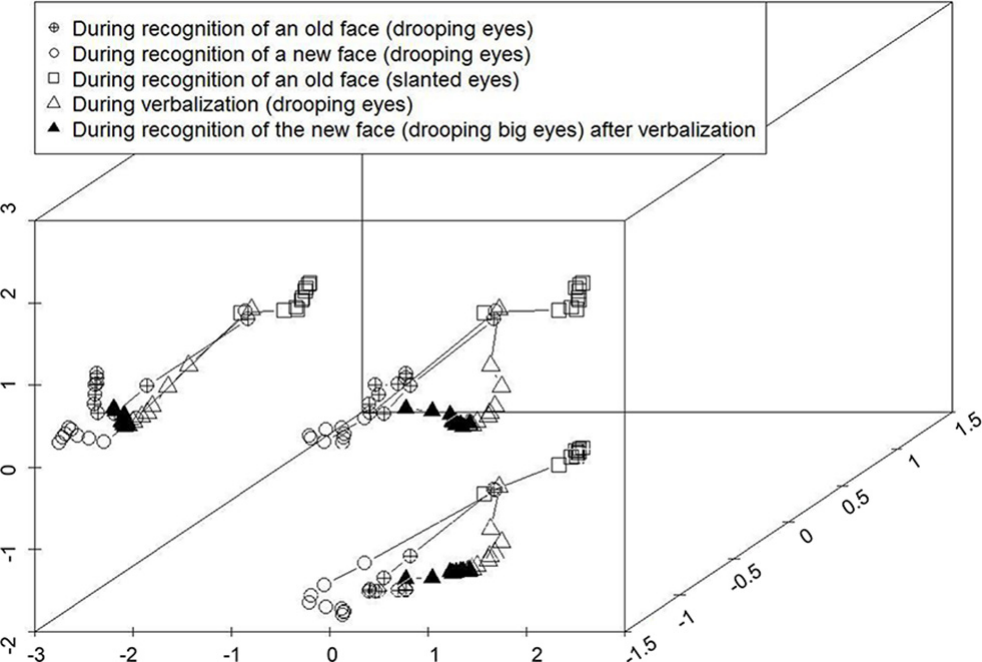
Multidimensional scaling analysis of the internal activities during settling in the face recognition task. Circular and square markers denote the face recognition activities without verbalization. The circular markers represent face recognition for two visually similar faces (“drooping eyes”) whereas the square markers represent that for visually dissimilar one (“slanted eyes”). Triangular markers denote activities during and after verbalization. To aid visual understanding, the 3-D plots were projected to the 2-D spaces (left and bottom planes).

Next, a multidimensional scaling analysis (MDS) was conducted to compress the multidimensional information represented in the hidden layer so that internal activities of the model could be visualized. Specifically, we measured hidden layer activities at every 10 time ticks when the network activities were settling down into the stable attractor state. Fig 4 shows this settling process for three representative items. Specifically, the filled and open circular markers represent face recognition for the old face with (thin) drooping eyes and a new face with (big) drooping eyes, respectively (see Fig 3A). As the proximity of these two plots shows, face recognition for these two visually similar cases involved highly similar internal activities. In other words, discriminating these visually similar faces required the computation of fine-grained internal representations. On the other hand, the plot of the squares, which represents the recognition for the old face with slanted eyes, is distant from the circle plots in the multidimensional space. This indicates that the settling process of the recognition for a visually dissimilar face required a dissimilar internal representation to compute. These suggest that, although their attractor states were unique for each item, similar inputs were associated with similar/neighboring attractor basins while dissimilar input fell into a dissimilar (distant) attractor basin. This acquired knowledge about the similarity structure was generalized to represent an untrained “new” face correctly, thus uniquely, in the visual image layer.

### Instantiation of verbalization process in the model

Once the model’s internal processes during face recognition were understood and visualized, the next step was to investigate the impact of verbalization on this internal activity. The verbalization procedure was instantiated to the model using the following procedure. First, some units in the verbal layer received an external input of 1.0, and the outputs of these verbal units were determined by the following equation:

output=initialoutput+0.5*(externalinput−initialoutput) (3)

Where initial output is a logistic function of the summed weighted inputs from other units (see prior Eq 1 Eq 2). Thus, this equation scaled the contribution of the external input and that of the internal activities (from the hidden layer) to unit activities (i.e., soft-clamping). Then, the activations spread from the verbal layer to the other layers, including the visual image layer, for 10 cycles. After 10 cycles, these external inputs to the verbal layer were removed or maintained, depending on the experimental conditions that were simulated (see later), and the model was presented as a retinotopic input. After this, the previous procedure for testing face recognition was applied.

An example outcome of this recognition after verbalization is shown in Fig 3B (lower half) and Fig 4 (triangle markers). As we mentioned above, the right column of Fig 3B shows the generated outputs in the visual image layer based upon the retinotopic inputs for two visually similar “old” and “new” faces. Although these two retinotopic inputs were the same examples as those in Fig 3A (upper half), these were confused with each other after verbalization (Fig 3B). Thus, external activation of three verbal units for drooping eyes, long-nosed, and for down-turned mouth made the model represent an old face in the visual image layer even though a new face was presented in the retinotopic layer. The internal activities during verbalization and during face recognition (after verbalization) of the untrained “new” face were plotted in the same multidimensional space (triangle markers in Fig 4). During verbalization (open-triangle markers), the hidden layer activities settled down near to the attractor basin of the visually similar, yet trained “old” face (i.e., filled-circle markers). Thus, even after the retinotopic input for a “new” face was presented, the internal representation did not move closer to its correct attractor (i.e., open-circle markers) but was captured by the wrong attractor basin (i.e., filled-circle markers). As a result, a confusion (false alarm) occurred.

Thus, crucially, the present model instantiated the core processing principles of the recoding interference hypothesis [2,3,5]. Namely, verbalization changed (recoded) the nature of representations, rather than shifting the types of processing (i.e., TIPS, [19]). Such a fully interacting system represented a face in a distributed manner, and such a distributed representation was affected (not shifted to another process-specific representation) by verbalization. Then, this recoded representation was used for (or affected) subsequent visual recognition, and resulted in a failure in computing item-specific information.

Before moving to the next analysis, it is worth emphasizing that in this simulation, a face recognition phase followed immediately after verbalization. In this sense, this phenomenon might be closely allied to a so-called priming effect [34]. It will be discussed later.

So far, we have demonstrated the model’s training performance, generalization ability, and we have visualized the change in the internal activities following prior verbalization. Next, in order to provide a more direct comparison with human data in the existing verbal overshadowing literature, “old”/“new” judgment-based recognition accuracy was measured in the model.

### Simulation 1. “Old”/“New” recognition judgment on the basis of polarity values

Given that most of the empirical studies on verbal overshadowing involved an “old”/“new” recognition judgment or an N-alternative forced choice task, it is worth measuring the corresponding performance of the model. For this purpose, the first step was to measure a variable that is known to affect “old”/“new” judgments of each item. One such a candidate is an item’s familiarity [35–37], as is assumed in a signal detection theory [37]. If it is possible to measure how familiar each item is to the model, then we can systematically assess the model’s “old”/“new” judgment on each item.

For this purpose, we measured how closely to the polar values (i.e., 0 or 1, hereafter *polarity*) a retinotopic face input activated the units in the visual image layer [38,39]. The rationale behind this polarity analysis is as follows: A unit activity near to one of the polar values means that the model is confident about what to do with that input, regardless of whether it is active or inactive. This is because some units are specifically tuned to presented stimuli during learning [40]. If some units respond more accurately to an item by chance, these activations are reinforced by a learning algorithm to respond more strongly to the item the next time it is presented. In contrast, other units are suppressed so as not to send noise. Thus, a network generates an output near to a polar value for trained, familiar items, whereas an output near to the middle of the (sigmoidal) range occurs for an untrained, unfamiliar item. In other words, whether activity is near to a polar value indicates how unambiguously each unit responds to the input, thereby forming an index of familiarity. Specifically, polarity for each unit was calculated using the following equation:

$$Polarity\;in\;unit_{i}=a_{i}*\text{log}\;2\left(a_{i}\right)+\left(1-a_{i}\right)*\text{log}\;2\left(1-a_{i}\right)+1$$ (4)

Where *a*_*i*_ refers to the activity of *unit*_*i*_. Thus, as the equation above shows, polarity ranges from 0 to 1. A high polarity value denotes that the unit elicits close to a 1 or 0 response (close to the asymptote of the sigmoid), whereas a lower polarity value denotes that the unit produces a response near 0.5 (i.e., close to the linear range of the sigmoid). If polarity distributions for all 32 “old” faces are higher than those for the “new” faces, then a criterion value can be set to optimize the “old”/“new” judgment accuracy to be above chance.

**Fig 5:**
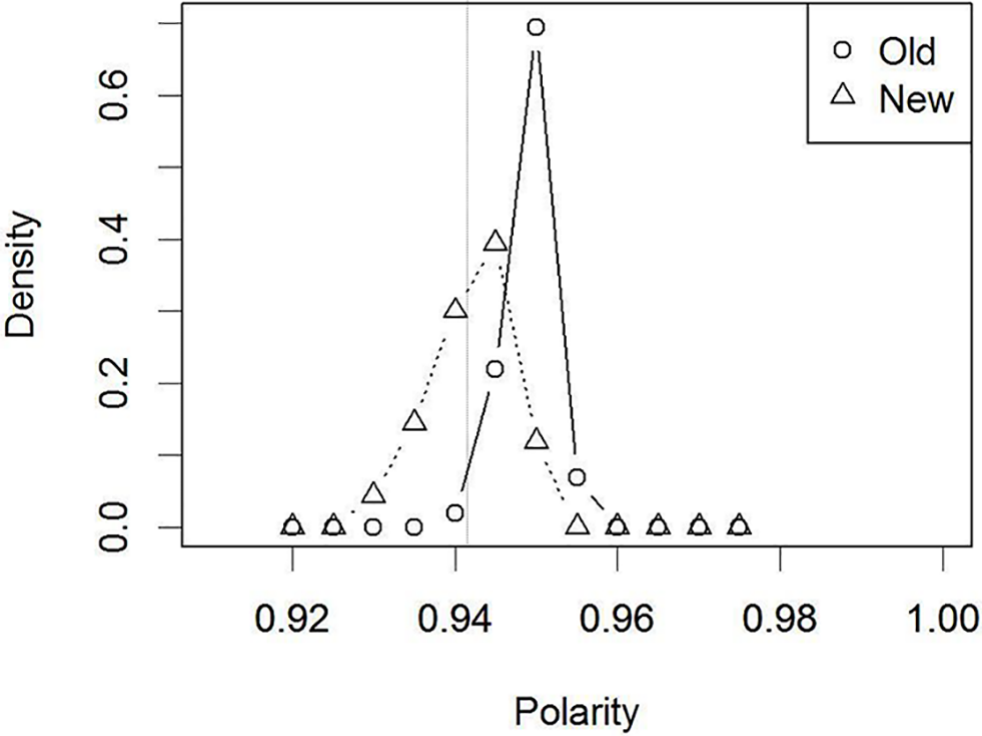
Distribution of polarity values for the “old” and “new” faces. Simulation 1 (without verbalization). The dotted vertical line indicates the decision criterion that produces “old”/“new” judgment performance comparable to humans.

Fig 5 shows the outcome of this analysis. Polarity values for each unit were measured on the 10^th^ cycle of a visual recognition trial, and were averaged across the units in the visual image layer. To produce a smoother distribution curve, the outcomes were averaged across five different models (each initiated with a different random status). In this figure, the polarity distribution of the 32 “old” faces (circular markers) is located to the right of the polarity distribution of the 32 “new” faces (triangular markers). The dotted vertical line at polarity value of 0.942 indicates the “old”/“new” decision criterion that produced comparable performance to humans. With this criterion, 100% of the “old” items were categorized as “old” (i.e., hits), whereas 30% of the new items were correctly categorized as “new” (i.e., correct rejections). Thus, the averaged “old”/“new” judgment accuracy resulted in 65% correct performance (SE = 0.02). This performance is compatible with normal human recognition accuracy, as reported in the verbal overshadowing literature (e.g., 64% correct, [1]). Note that human accuracy refers to the proportion of the participants who correctly chose the target face in a single-trial test. Thus, the polarity analysis also suggests that the model was successfully trained for face recognition in terms of “old”/“new” recognition, based on the polarity values.

#### Testing the effect of verbalization on the “old”/“new” decision on the basis of polarity

The next series of analyses concerned whether verbalization changes the polarity distributions in Fig 5, in comparison to the control condition (i.e., without verbalization). Overlapping of the two distributions to a greater extent suggests a lower “old”/“new” judgment accuracy. In contrast, if the distributions remain unchanged or overlap less, this suggests that recognition performance would remain unchanged or even improve (i.e., no deleterious effect of verbalization).

The verbalization procedure was instantiated via the same procedure as mentioned above (10 cycles after the external input to the verbal layer). The polarity values were measured at the 10^th^ cycle after a retinotopic input. A subsequent series of simulations aimed to reproduce the human data on verbal overshadowing produced from various experimental paradigms, by manipulating (1) which verbal units to activate, and (2) when to remove the external verbal inputs.

### Simulation 2. Target-distractor similarity: Similar condition

First, we examined whether the model was able to reproduce standard verbal overshadowing. For this purpose, we instantiated the experimental situation where verbal overshadowing is most readily observed (see Introduction). Several studies have suggested that participants are more likely to fail target recognition with a similar distractor or a typical face [6,7]. Such a high similarity condition was instantiated in the model here. Specifically, if the target and distractors are similar to each other, a logical consequence is that human participants must have verbalized information which is plausible (i.e., consistent) for *both* the target and distractors before recognition. Therefore, a simulation of these human experiments involved the external activation of “correct” (i.e., consistent with the to-be-judged retinotopic input, be it “old” or “new”) verbal units before presentation of a retinotopic input. This external verbal input was maintained even after the retinotopic input presentation, as it was natural to assume that the participants “believed” these verbalized features were correct, even during a face recognition test.

**Fig 6:**
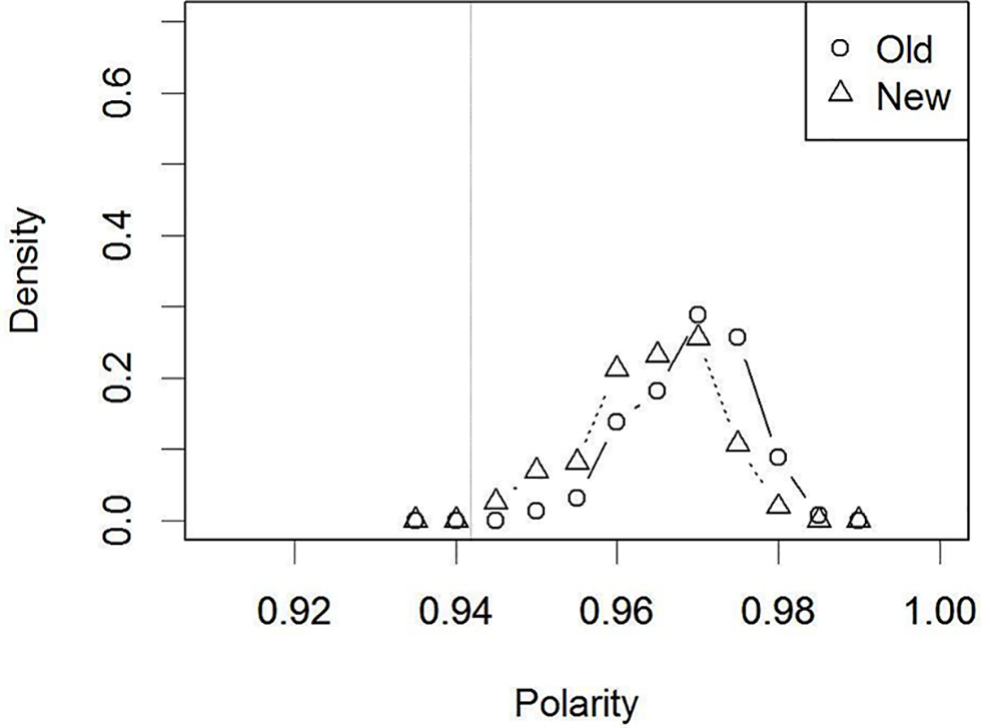
Distribution of polarity values for the ‘old’/‘new’ faces in Simulation 2 (after verbalization): High target-distractor similarity. The dotted vertical line indicates the decision criterion that was set in the control condition (Fig 5).

Fig 6 shows the resultant polarity distributions. Verbalization moved the polarity distributions such that the “old” and “new” distributions overlapped to a greater extent. That is, the model could not discriminate the “old” faces from “new” on the basis of polarity values (i.e., familiarity). Indeed, the “old”/“new” decision with the same criterion value as the control condition (i.e., Fig 5, without verbalization) resulted in lower accuracy (50% correct, SE = 0, *t* (4) = 6.69, *p* = .002). Thus, the model successfully reproduced standard verbal overshadowing.

### Simulation 3. Target-distractor similarity: Dissimilar condition

**Fig 7:**
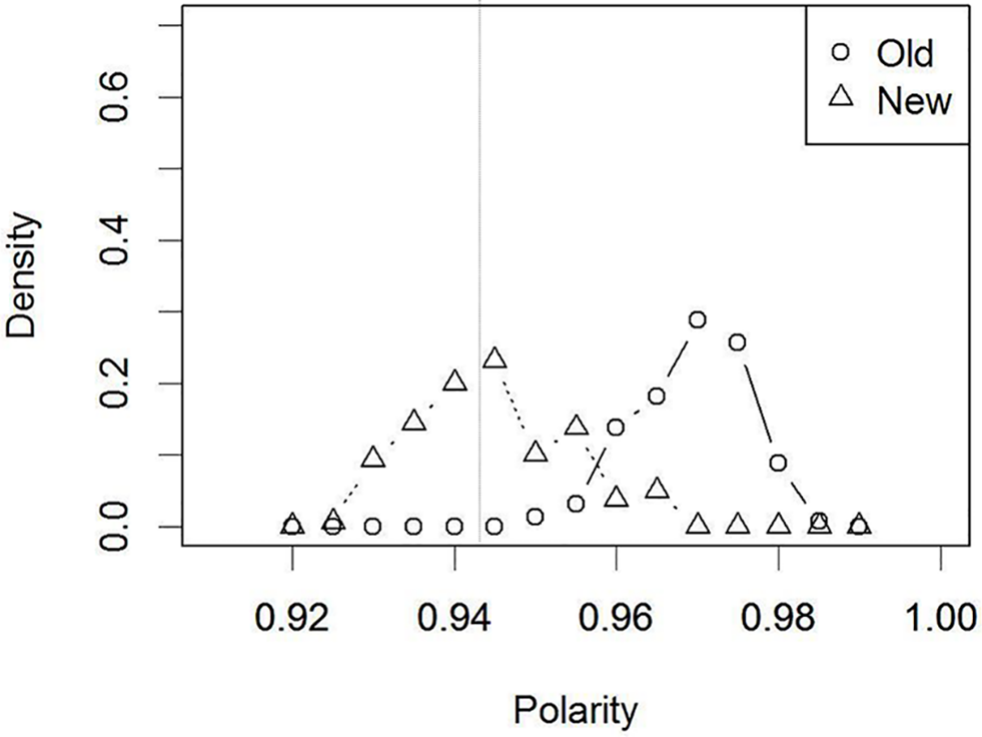
Distribution of polarity values for the ‘old’/‘new’ faces in Simulation 3 (after verbalization): Low target-distractor similarity. The dotted vertical line indicates the decision criterion that was set in the control condition (Fig 5).

Next, it is known that verbalization does not impair visual recognition when the dissimilar distractors or atypical faces were presented [6,7]. Under such experimental situations, it is natural to assume that the generated verbal descriptions should be correct/consistent with the target appearance, but less so with the distractors. Therefore, a simulation of these experimental situations involved the external activation of the consistent verbal units as the retinotopic input when testing an “old” face. In contrast, the inconsistent verbal units were externally activated before presenting an untrained “new” retinotopic input. The resultant polarity distributions are shown in Fig 7. These distributions were separated to a greater extent than the control condition (without verbalization in Fig 5). Indeed, “old”/”new” judgments with the same criterion as the control condition did not reveal a detrimental effect of verbalization (66% correct, SE = 0.06, *t* (4) = -0.11, *p* = .914, *n*.*s*.), consistent with human experiments.

### Simulation 4. Reproduction and understanding of counterevidence against the recoding interference hypothesis: Verbalization of an irrelevant face

As we demonstrated by the MDS analysis (Fig 4), the present model instantiated the core processing principles of the recoding interference hypothesis. Therefore, it is of utility in accounting for the experimental phenomena that weigh against the recoding interference hypothesis.

**Fig 8:**
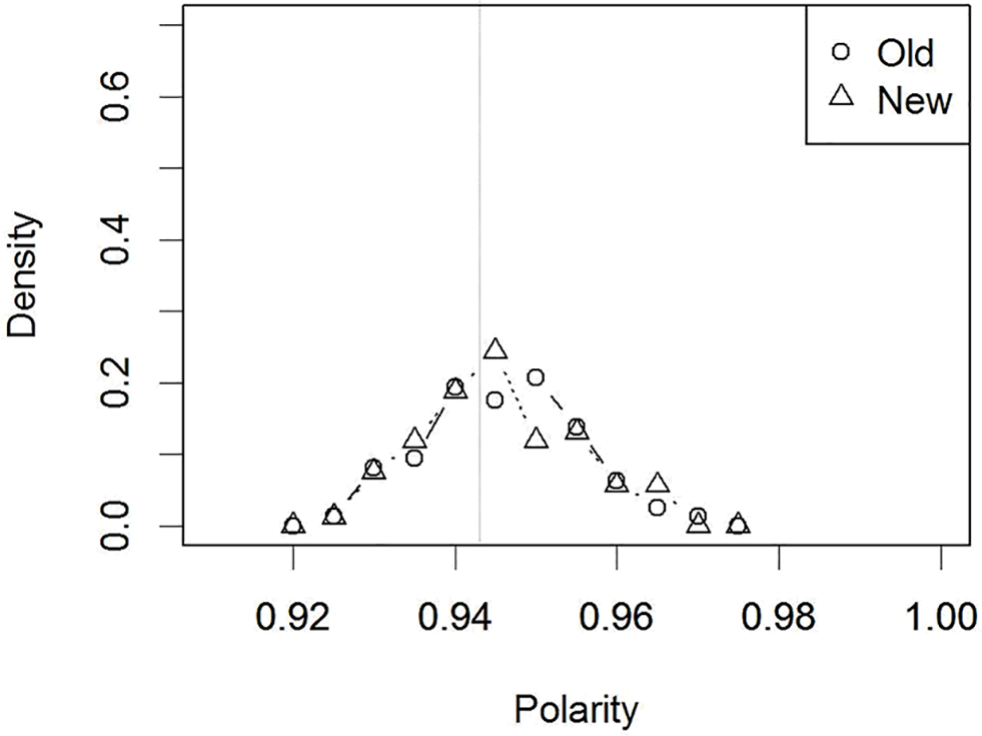
Distribution of polarity values for the ‘old’/‘new’ faces in Simulation 4 (after verbalization): Verbalization of an irrelevant face. The dotted vertical line indicates the decision criterion that was set in the control condition (Fig 5).

The first target was the effect of verbalizing an irrelevant face (e.g., [23]). Unlike the simulations so far, the external verbal inputs were not consistent with the features of the retinotopic face input. This is equivalent to human experiments where individuals were asked to describe an unrelated face before recognition. In addition, it is natural to assume that individuals in such experiments do not believe their descriptions are relevant to the target features. In order to reflect this assumption in a simulation, the external input to the verbal layer was removed when a retinotopic input was presented. The resultant polarity distributions are shown in Fig 8. In this case, the distributions for the “old” and “new” faces were more coincident than in the control condition (Fig 5). It follows that the “old”/”new” decision performance declined (*M* = 50% correct, SE = 0.03, *t* (4) = 6.55, *p* = .002) when evaluated with the same criterion as the control condition, a signature of verbal overshadowing. Thus, it is unnecessary to incorporate a modular process into a theory, such as a “shift of processing” (e.g., TIPS). Even without such an additional mechanism, one can account for the detrimental effect of verbalizing an unrelated face (e.g., [23]).

### Simulation 5. Reproduction and understanding of counterevidence against the recoding interference hypothesis: Relationship with verbal description accuracy

Another piece of counterevidence against the recoding interference is that some studies have failed to detect a significant correlation between description accuracy and the effect size of verbal overshadowing [1,6,8,25]. Our hypothesis is that this detection failure may stem from a possible confound with how accurately the verbal descriptions capture the *distractor* faces, not just the target face, only the latter of which has been investigated in existing literature. Indeed, our simulations so far have already demonstrated this idea. Specifically, Simulation 2 showed that correct description of distractors declined recognition accuracy even though the description accuracy for the target face was perfect. Therefore, this demonstration indicates the necessity to control for or to systematically investigate the effect of this *distractor description accuracy*, which refers to how closely the descriptions of the target coincidentally capture distractor faces. As mentioned in the Methods, the model represented a face in terms of three types of verbal information (eyes: drooping/slanted, nose: long/button-shaped, mouth: thick/downturned). Thus, it was possible to instantiate four levels of verbal description accuracy (i.e., 0%, 33.33%, 66.66%, or 100%) by activating from 0 to 3 of the verbal units correctly.

**Fig 9:**
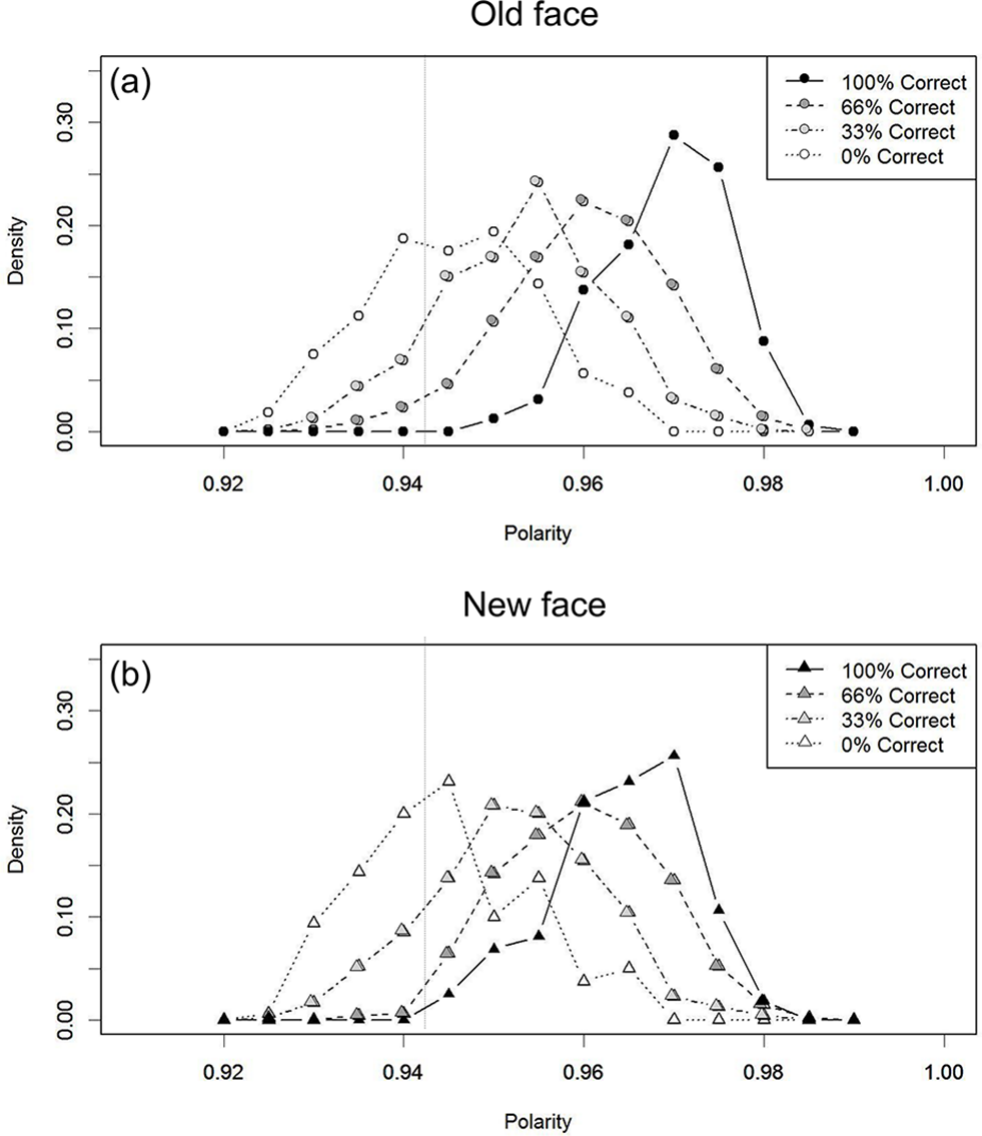
Polarity distributions as a function of description accuracy for (a) “old” and (b) “new” faces. The dotted vertical line denotes the criterion that was set in the control condition (Fig 5).

Fig 9 shows the polarity distributions for the old faces (a) and new faces (b) as a function of how accurately/consistently the externally activated verbal units captured the retinotopic input. Clearly, both distributions moved towards the left (i.e., lower polarity) as description accuracy decreased. Crucially, a series of comparisons between one of the distributions in Fig 9A (“old” faces) and one in Fig 9B (“new” faces) demonstrated a *non-significant correlation* between target description accuracy and the “old”/“new” recognition judgment performance, as is occasionally observed in human experiments [1,6,8,25]. Specifically, we will describe three cases in descending order of target description accuracies, where the magnitude of verbal overshadowing did not necessarily decline in parallel. As a first case, imagine that the verbal descriptions captured the target face features perfectly (i.e., the rightmost distribution in Fig 9A). In this situation, if these descriptions happened to capture the face features of the distractors perfectly (i.e., the rightmost distribution in Fig 9B), “yes”/“no” judgment performance declined (50% correct accuracy, see Simulation 2), a signature of verbal overshadowing. Next, imagine the target description captured 66.6% of the target face correctly (the dark gray marker in Fig 9A). In this situation, if these descriptions happened to capture only 33.3% of the distractor faces correctly (i.e., the light gray marker in Fig 9B), then the two distributions did not overlap as much as in the first case (above), which means that the judgment accuracy declined to a lesser extent (54% correct, SE = 0.01, *t* (4) = 4.57, *p* = .01). That is, the second case showed a smaller effect of verbal overshadowing than the first case, despite the lower target description accuracy. Finally, a third case was more critical. If we compare the distribution of the light gray markers in Fig 9A (i.e., 33.3% accurate) with the leftmost distribution of Fig 9B (i.e., 0% accurate for “new” faces), again the two distributions do not overlap greatly, indicating no decline in recognition accuracy (62% correct, SE = 0.05, *t* (4) = 0.67, *p* = .537). Therefore, the last case showed the highest recognition accuracy despite having the lowest target description accuracy of the three cases. Taken together, these three cases illustrate that recognition accuracy did not decline in parallel with target description accuracy.

As is clear from these analyses, the crucial factor was not only target description accuracy but also distractor description accuracy. The more accurately the generated verbal descriptions happened to capture the distractor faces correctly, the more the polarity (familiarity) distribution for distractors is shifted rightward.

### Simulation 6. Explaining the “unreliability” of verbal overshadowing in terms of individual differences in subjects/items

Finally, one of the key issues in verbal overshadowing is the occasional replication failures in the literature, although the recent large-scale replication project has successfully demonstrated a significant effect of verbalization [4]. Therefore, we aimed to clarify what modulates the detectability of this phenomenon, focusing on two factors, namely individual differences among subjects and those among items.

**Fig 10:**
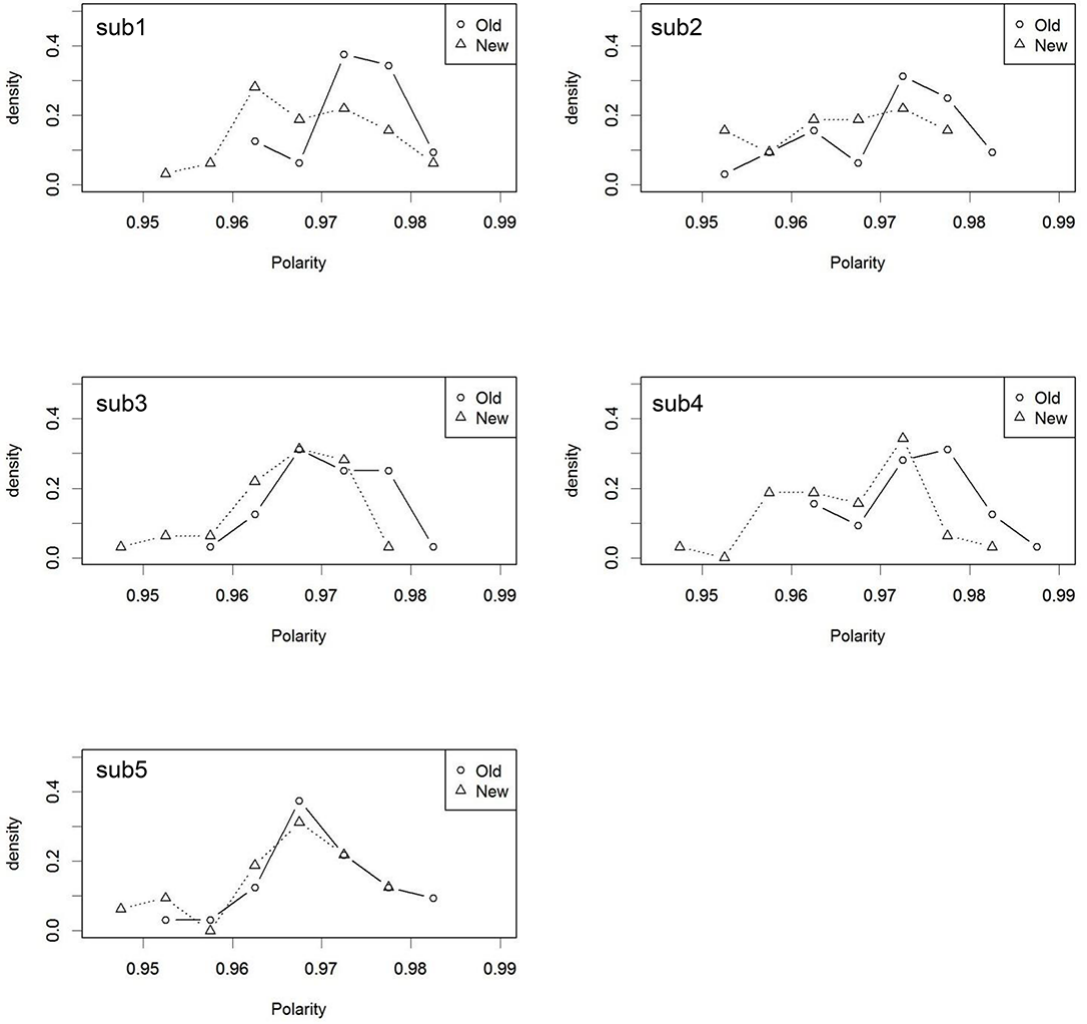
Individual model polarity distributions for the “old”/“new” faces in the similar target-distractor condition. The average of these 5 models corresponds to Fig 6 (Simulation 2).

First, as mentioned above, Fig 6 showed the polarity distributions for the trained “old” and for the untrained “new” faces, *averaged* across five different models (with different initial status) when the target-distractor similarity was high. However, once we split this averaged histogram (Fig 6) into individual models (Fig 10), then clear individual differences were observable. Specifically, the two polarity distributions did not overlap for some of the individual models. Thus, this simulation demonstrates that verbal overshadowing is sensitive to individual differences.

The final analysis involved differences among individual items. So far, we have shown two polarity histograms (distributions) for *all* 32 trained (“old”) and *all* 32 untrained (“new”) faces, and then estimated the model’s “old”/“new” judgment performance based on these distributions. However, a standard verbal overshadowing experiment involves a single-trial testing procedure. It is possible to instantiate such a single-trial testing procedure in the present model and to observe the outcome. Specifically, imagine one item (target) is randomly chosen from the polarity distribution of the trained “old” items (e.g., Fig 10). Similarly, N items (distractors) are randomly chosen from the polarity distribution of the untrained “new” items in the same figure (Fig 10). As a result, it is unsurprising that the polarity value of one of the randomly-selected “new” items is higher, by chance, than the single “old” item. That is, a single-trial testing procedure in the model is quite sensitive to differences in individual item polarity. Thus, unless the polarity distributions for the “old” and “new” faces do not overlap at all, a familiarity-based “old”/“new” decision performance can be noisy in a single-trial testing procedure.

## Discussion

The present study used a computational modeling approach to understand the mechanism by which verbalization of non-verbal memory affects subsequent non-verbal recognition performance, an effect known as verbal overshadowing. So far, there are two primary explanations of verbal overshadowing: the recoding interference hypothesis and the transfer-inappropriate processing shift (TIPS) hypothesis. The key difference between these hypotheses is whether an operation-specific representation is postulated or not.

The present model instantiated the core processing principles of the recoding interference hypothesis rather than TIPS. Indeed, verbalization in the model changed the pattern of activations in the internal layer (Fig 4), which in turn impaired the subsequent computation of the item-specific representation of a retinotopic face input (Figs 3B and 4). Thus, an implemented computational model allows an explicit instantiation of one theory without ambiguity, and can advance the understanding of how cognition emerges from the model.

### Computationally grounded recoding interference hypothesis for verbal overshadowing

The present model successfully simulated standard verbal overshadowing (Simulations 1–3), and the detailed analysis of the internal representations (Fig 4) demonstrated that accuracy was reduced by the mechanism assumed by the recoding interference hypothesis. More crucially, such a model also reproduced the experimental phenomena that had been advocated as “counterevidence” against the recoding interference hypothesis (Simulations 4–5). These phenomena included the fact that verbalization of a non-target face impairs recognition of a target face (e.g., [23]) and the fact that the accuracy of verbal descriptions does not linearly explain its negative effect on subsequent face recognition [1,6,8,25]. These demonstrations mean that these phenomena are predictable from the recoding interference hypothesis, contrary to prior expectations.

An explicit implementation of a computational model aims to advance the understanding of a cognitive behavior, not to merely reproduce it. Therefore, it is crucial to explain how these phenomena emerge from the implemented model. First, by verbalizing irrelevant information (e.g., non-target face), the network settles down into an attractor that captures information irrelevant to the target face (S3 Fig). That is, given that the attractor states in the hidden layer captured the visual similarity of inputs (see Fig 4), the resultant internal representation by verbalization was dissimilar from that required for recognition of the target face. As a result, the network was captured by a distant attractor state, and it was difficult to travel into the correct attractor basin in order to compute target-specific information. Thus, verbalization induces noise in the hidden layer activities. In such a situation, the “old” items lose an advantage of training, and the resultant polarity distributions for the “old” and “new” items overlap to a greater extent, thereby reducing the “old”/“new” judgment accuracy.

Second, the present model also provides an account for why target description accuracy does not linearly predict recognition accuracy. If we focus on only target description accuracy and the polarity values for “old” faces only, then certainly these two correlate with each other (i.e., Fig 9A). However, Simulation 5 demonstrated that the generated verbal descriptions affected not just the polarity (familiarity) values of “old” items but also those of “new” items, as a function of how accurately the descriptions captured the distractor faces (Fig 9B). This was the reason why target description accuracy in isolation does not necessarily predict the effect size of verbal overshadowing in a linear fashion. The past experimental studies have not considered the role of distractor description accuracy.

### Distributed representation of a fully interactive system vs. operation-specific representation in each of multiple modules for face recognition

Studying verbal overshadowing is not only for the sake of this phenomenon. Rather, an investigation of the mechanism in this domain has implications for other domains as well. Indeed, the controversy between the recoding interference hypothesis and the transfer-inappropriate hypothesis mirrors the controversies between a single-system account (distributed cognition) and a multiple-systems account (each system has its own processing-specific computational principle/representation) in various other domains, such as word reading [41,42], word recognition [39,42], priming effects [38], past-tense inflection [43,44], and action sequences [45,46]. In all of these domains, some behavioral data were advocated as counterevidence against a single-mechanism account. Indeed, it is not surprising that any theory can explain a phenomenon if the number of processing principles to assume is a free parameter. Such a multiple-systems account sacrifices the parsimony of a theory, and sometimes it is only informally providing a model (i.e., only words or boxes-and-arrows). The computationally implemented parallel-distributed processing models listed above (single-system accounts) have successfully reproduced such “counterevidence,” thereby providing more parsimonious accounts.

The history of verbal overshadowing has partially followed this same path. Thus, some experimental findings have been more easily explained by increasing the number of processing principles, each of which operates independently (TIPS: [19]), but it has never excluded the possibility that these phenomena can be underpinned by a fully-interactive system with distributed representations. The present study actually demonstrated this idea. Thus, there is no need to assume differential processing principles or operation-specific representations. This way, the present investigation can be located in the context of a broader controversy. Related to this, it is worth reiterating that the computational mechanism of verbal overshadowing we demonstrated (Fig 4) is consistent with priming effects that past PDP models have demonstrated [38]. Indeed, a recent large-scale replication study has demonstrated that verbal overshadowing is robustly replicable if face recognition immediately follows verbalization [4]. This procedure was exactly instantiated in the current model. Thus, our model prompts a future experimental study that will more directly test whether the effects of verbalization and those of priming are based on a common mechanism. In this way, verbal overshadowing will be located in the context of broader psychological issues.

In addition, the current study is relevant to face recognition itself, not just verbal overshadowing. As mentioned in the introduction, our model links the assumption of the verbal code and visual code, like Bruce and Young’s [15] face recognition model. A key issue is how interactive/modularistic these two are. Thus, in this context, an issue is whether face recognition is grounded in a distributed processing system, whose representation is also used in other domains such as language, or not. On this issue, neuroscience data have been advocated as a support for a modularistic view of face recognition. For example, the fusiform face area (FFA) in the right hemisphere is known to be activated to a greater extent when recognizing a face rather than other stimulus types [47–49]. Furthermore, damage in this area sometimes gives rise into a face-specific recognition impairment without reducing recognition performance for words [50]. Such a dissociation is readily explained by a multiple-module view that assumes a language-specific processing system (and its processing-specific representation) and a face-specific processing system independently. However, Plaut and Behrmann [27] instantiated a single-mechanism account into a computational model, in which representations were distributed across the layers within the model, and all of the units, links, and activations in the model contributed to recognition of all the stimulus types (faces, words, and houses). The present model targeted a more specific phenomenon, namely verbal overshadowing, but our model is clearly a variant of Plaut and Behrmann’s [27] face recognition model. That is, the present study extended the plausibility of Plaut and Behrmann’s [27] single-account theory of face recognition to verbal overshadowing. Given that verbal overshadowing is crucially relevant to daily life and legal issues (e.g., eyewitness testimony), the present study bridges the broad theoretical domain and the more applied domain within the same framework.

### Applied issues

Verbal overshadowing has been vigorously discussed in terms of relevance to applied issues. There are two unique contributions from the present study in this regard. First, one should not ignore the quality (accuracy) of verbal descriptions that have been generated before the recognition phase. To date, consensus has been that description accuracy does not necessarily correlate with the degree of recognition impairment [1,6,8,25]. Such a conclusion would undermine the importance of analyzing the verbal descriptions. However, we demonstrated that verbal descriptions certainly predicted recognition impairment in a complex manner. Specifically, when evaluating the credibility of a testimony, the foils should be carefully selected in terms of their relevance to the generated verbal descriptions, and the generated verbal descriptions should be regarded as including potentially crucial information. Second, the present study confirmed the accountability of the recoding interference hypothesis [3,5]. That is, the current study indicates that any theory about *interference* in the memory domain is potentially useful when discussing the effect of verbalization on non-verbal recognition, both theoretically and practically.

### Replicability

As mentioned above, there have been occasional failures to replicate verbal overshadowing, although the recent large-scale replication project demonstrated its robustness [4]. Therefore, it would be constructive to clarify the reasons for the occasional replication failures. In addition to the target-distractor similarity factor (e.g., [6,7]) and the description accuracy [2,5,8], the present study also clarified the effect of other random factors. Thus, there were individual differences in subjects regarding how the familiarity distribution of old items and that of new items change by verbalization (Fig 10). In addition, the present model demonstrated how noisy (i.e., affected by individual item differences) a single-trial testing method is. One may find such a conclusion unsurprising, but, it is illustrative to explicitly demonstrate the effects of these random factors in a computational model, rather than stating a mere verbal explanation. Taken together, we suggest that it is premature to argue whether verbal overshadowing exists or not, in an all-or-none manner. Indeed, several studies which involved a multiple-trial testing method had a good replicability [17,18,51]. Also, it is worth mentioning that although these studies are referred as collateral evidence for TIPS, our model shows no necessity of implementing a modularistic system/process like a TIPS.

### Other experimental studies

Finally, the limitations of the current study should be acknowledged to prompt future studies. In a strict sense, the present model did not cover all of the behavioral phenomena relevant to verbal overshadowing. For example, we did not consider whether our account can be extended to the visual imagery domain beyond the face recognition (e.g., [52]). However, we suggest that even if the same procedure (i.e., verbalization) reduces accuracy in two (or more) different non-verbal domains, there is no need to explain such decreased accuracy by a single theory. Unless there is a strong a priori reason to explain two phenomena by a single account, it would be more conservative to develop a theory within a domain. Our model also did not examine other effects found in verbal overshadowing literature such as verbal facilitation effect [2,33,53,54], and other similar phenomena such as a Navon effet [55,56]. In future studies, it should be examined whether the model can be extended to cover these phenomena. In addition, the present model should be added more realistic features of human face recognition. For example, the present model can describe limited facial information. If the model were permitted to describe a multiple of information, the model may have a better chance capturing correlation between incorrect description and identification accuracy [2]. Furthermore, it might be worth to examine how verbalization affects face recognition when a model implements more realistic facial input (i.e. input of facial features are updated continually by visual search). We made the present model as simple as possible for first step. We would like to emphasize that by presenting this model, we are providing a platform where other researchers can freely tackle these issues.

## Supporting Information

S1 Fig Visual images of 64 faces. (TIF)

S2 Fig Visual images of 64 faces with Gaussian noise. (TIF)

S3 Fig Multidimensional scaling analysis of the internal activities during verbalization of an irrelevant face and recognition of a target face. (TIF)

S1 File Simulation data set. (ZIP)
