# Supplementary figures and images for: Why Verbalization of Non-Verbal Memory Reduces Recognition Accuracy: A Computational Approach to Verbal Overshadowing
Source: PLoS One. 2015 Jun 10;10(6):e0127618. doi: 10.1371/journal.pone.0127618 (PMC4464652; doi:10.1371/journal.pone.0127618)

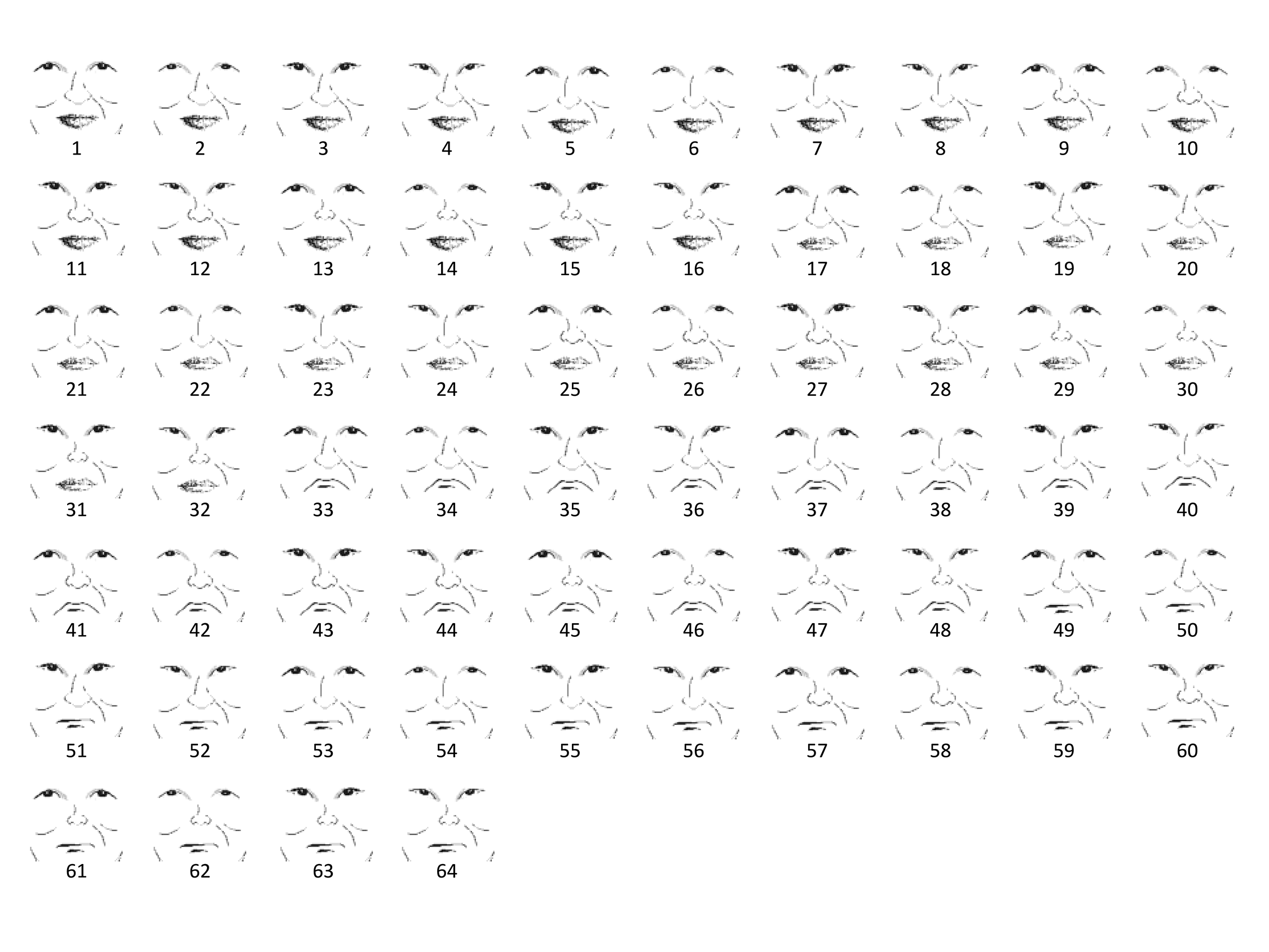

Supplement: S1 Fig — (TIF) [file pone.0127618.s001.tif]

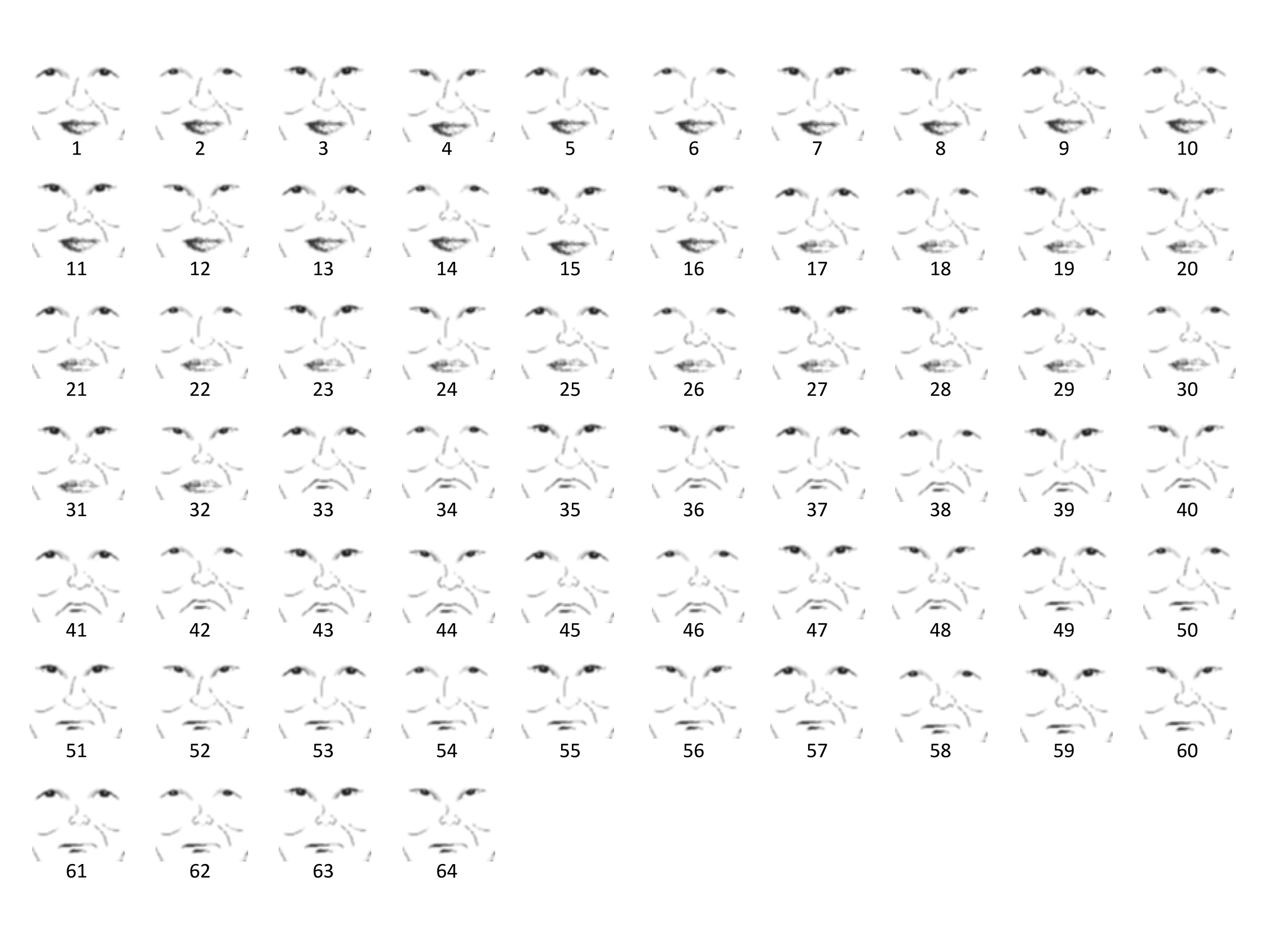

Supplement: S2 Fig — (TIF) [file pone.0127618.s002.tif]

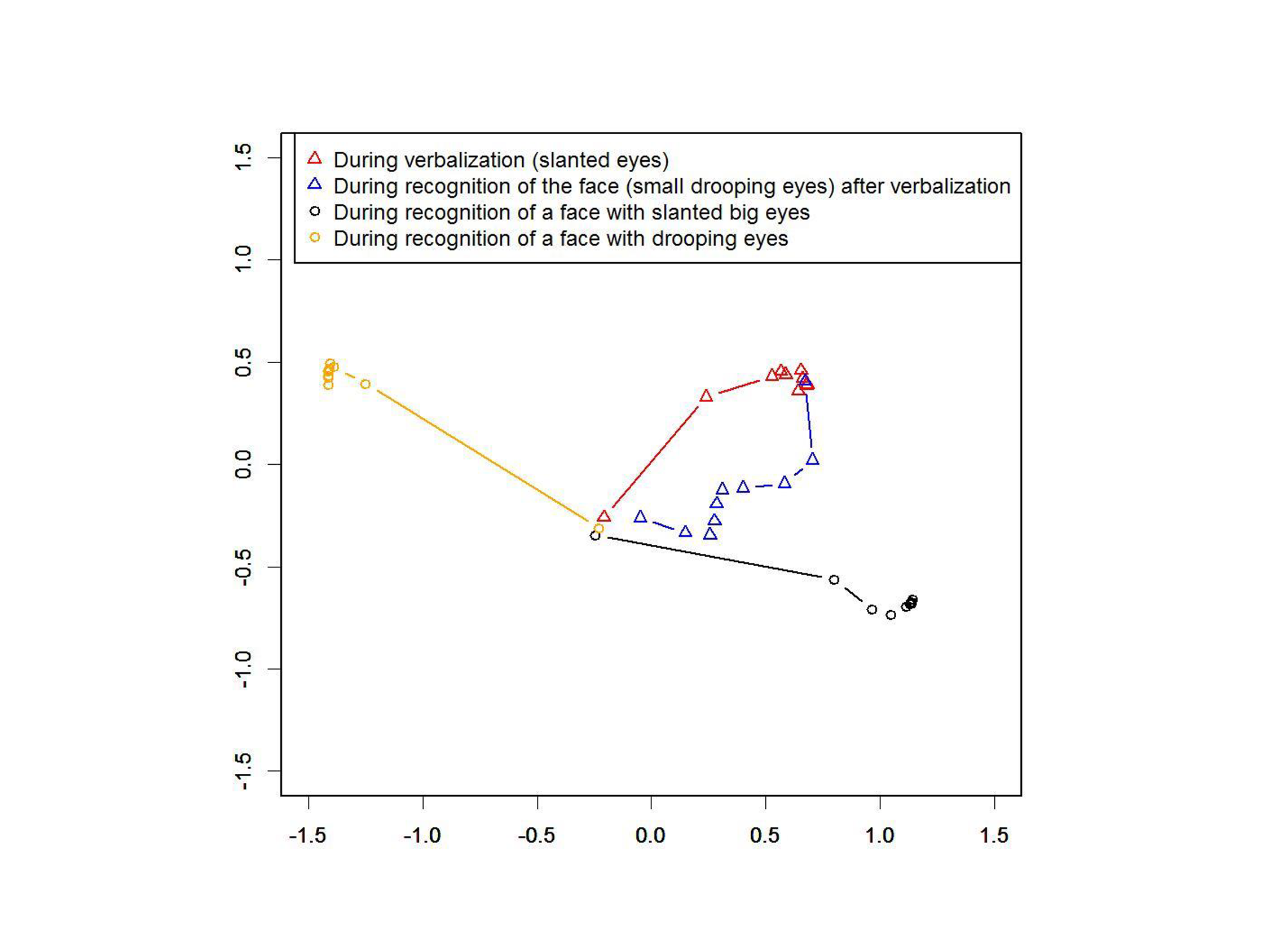

Supplement: S3 Fig — (TIF) [file pone.0127618.s003.tif]

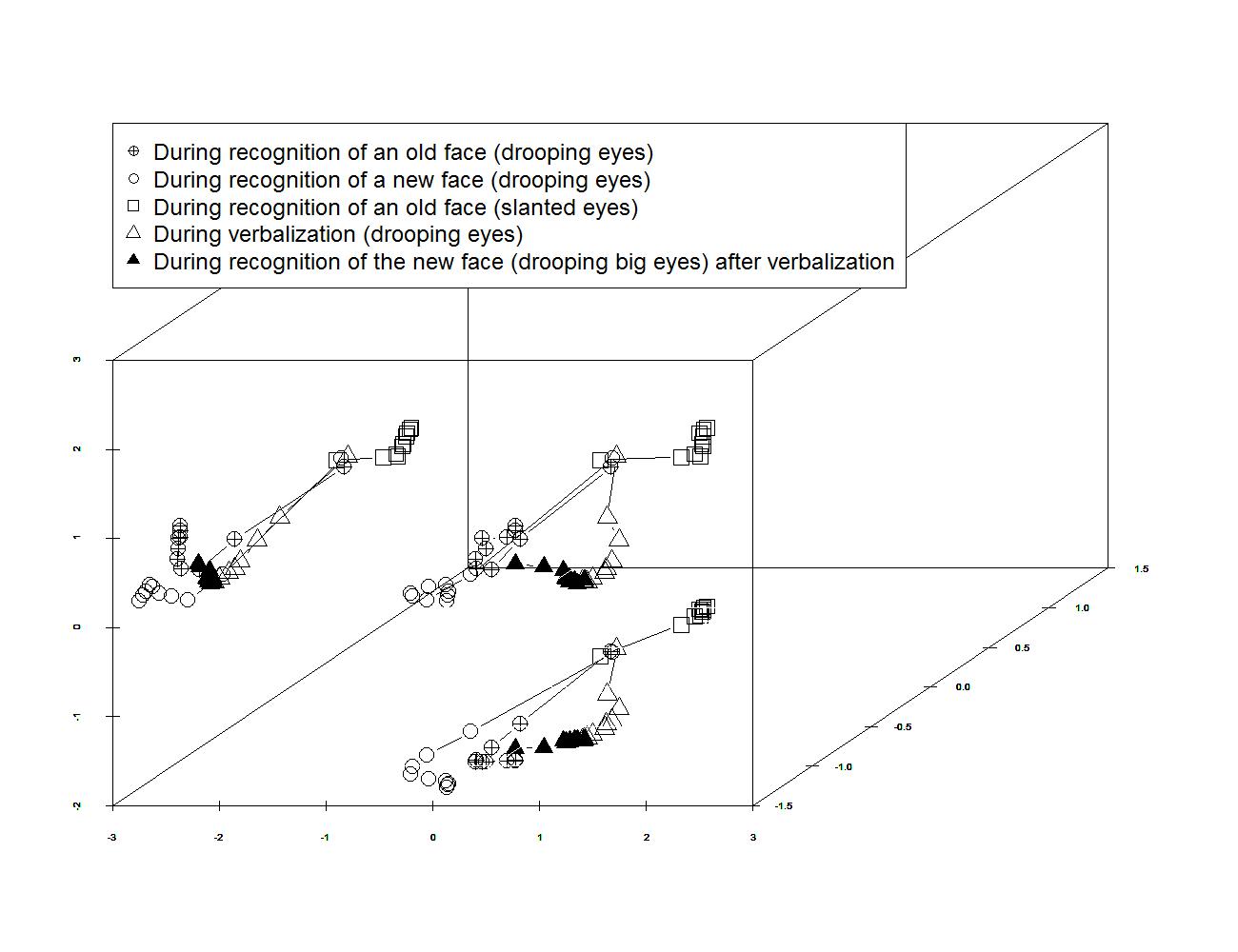

Supplement: S1 File — (ZIP) [file pone.0127618.s004.zip › PlosOne_simulation_data/MDS_Data/Figure4.jpeg]
